# Acknowledgment to Reviewers of *Antibodies* in 2021

**DOI:** 10.3390/antib11010009

**Published:** 2022-01-26

**Authors:** Antibodies Editorial Office

**Affiliations:** MDPI AG, St. Alban-Anlage 66, 4052 Basel, Switzerland

Rigorous peer-reviews are the basis of high-quality academic publishing. Thanks to the great efforts of our reviewers, *Antibodies* was able to maintain its standards for the high quality of its published papers. Thanks to the contribution of our reviewers, in 2021, the median time to first decision was 20 days and the median time to publication was 79.5 days. The editors would like to extend their gratitude and recognition to the following reviewers for their precious time and dedication, regardless of whether the papers they reviewed were finally published:
Ailin WangJurgen SanesAkiyoshi TakamiKalra Rajkumar SinghAlessandra VillaKarin HufnaglAli R. JazirehiKatja KlauszAlicja Maria Sochaj-GregorczykKirstie BertramAnastasia SveshnikovaKoichi KatoAndrew ScottLina BaranauskieneAngelica BiancoLuis Alvarez-VallinaAnn WhiteMaciej WłodarczykAnshu AgrawalMahendra DeonarainAnton LennikovMaria Antonietta CiardielloAntu K. DeyMarta KarazniewiczArne SkerraMd Kausar AlamBas De LaatMichal ZmijewskiBehnoush Abedi ArdekaniMichele BernasconiBin ZhanMing-Ching HsiehBingchun ZhaoNaga Venkata Sabarinath NeerukondaCatalina FlorezNicolae GicaChichun HoNima TaefehshokrChihyang WangNuria Álvarez-SánchezChristiane StadlerOleksandr KonievChuan ChenOxana SerovaClaudia KitzmüllerParameswaran RamakrishnanCollynn WoellerPaulina ŻarnowiecCory L. BrooksPhilippe BillialdDavid BrandonPierre RougéDavid VancePio ContiDebmalya BarhPriyanka KhareDennis R. GouletQuirina Santos-CostaDhaval K. ShahRatnadeep MukherjeeDidier BoquetRobert H. CarnahanDiego MoricoliRonald P. TaylorDominic NarangRoss ChelohaEduardo Ruiz-LópezRossella SartoriusEls VerhoeyenRyo MisakiFrancesco GiansantiScott DessainFrank KrauseShyh-Chyi LoGiada Maria VecchioSiva Krishna AngalakurthiGillian LoweStefanie K. WculekGiulia PezzoniStefano AngiariGordana Wozniak-KnoppSukmook LeeGregory IppolitoSushobhana BandyopadhyayGuangai XueSwati JainHarald KolmarSylvie BouvierHongnan CaoTae Hyun KangHuub SchellekensTal Noy-PoratIgnazio CaruanaTaru S. DuttJarmila CelakovskaTilman SchlothauerJeffrey V. LeytonTobias BerglerJohann BauerTomoyuki IgawaJohn De KruifTsvetelina VelikovaJordi Martorell-MarugánUtkarsh H. AcharyaJoseph KauerWei Sun


